# Association Between Glycated Hemoglobin and Severity of Coronary Artery Disease in Type 2 Diabetic Patients With Myocardial Infarction

**DOI:** 10.7759/cureus.81521

**Published:** 2025-03-31

**Authors:** Amna Rashid, Muhammad Salman Saeed, Mohamed Arshad Ghouse, Arman K Kunju, Nuh Umar, Abdul Eizad Asif, Bisma Naveed, Haiqa Asif, Muhammad Rafay Khan, Areeba Soaib

**Affiliations:** 1 Internal Medicine, Jinnah Hospital, Lahore, PAK; 2 Critical Care Medicine, Basildon University Hospital, Mid and South Essex NHS Foundation Trust, Basildon, GBR; 3 Internal Medicine, Nawaz Sharif Medical College, Gujrat, PAK; 4 Neurology, University Hospital Coventry & Warwickshire, Coventry, GBR; 5 Emergency Medicine, Basildon University Hospital, Mid and South Essex NHS Foundation Trust, Basildon, GBR; 6 Neuroscience, University Hospital Coventry & Warwickshire, Coventry, GBR; 7 Gastroenterology, Barts Health NHS Trust, London, GBR; 8 Internal Medicine, Shalamar Medical and Dental College, Lahore, PAK; 9 Cardiology, Jinnah Hospital, Lahore, PAK; 10 Internal Medicine, Rashid Latif Medical and Dental College, Lahore, PAK; 11 Acute Medicine, Basildon University Hospital, Mid and South Essex NHS Foundation Trust, Basildon, GBR

**Keywords:** artery, association, coronary, diabetes, disease, hba1c, infarction, myocardial, severity

## Abstract

Background

Cardiovascular disease is a leading cause of morbidity and mortality worldwide, with a high prevalence in developing countries, and coronary artery disease (CAD) is a major contributor to it. Diabetes mellitus is a major risk factor for CAD. However, research on the association between glycated hemoglobin (HbA1c) levels and severity of CAD is lacking in developing countries like Pakistan. Therefore, this study aimed to investigate the association between HbA1c levels and the severity of CAD in type 2 diabetic patients with myocardial infarction (MI).

Method

This cross-sectional study was conducted on 225 type 2 diabetic patients with CAD at Cardiology Unit, Jinnah Hospital, Lahore, Pakistan during the period of one year from August 2020 to August 2021. The recruitment of patients was done via consecutive sampling and predefined inclusion and exclusion criteria. Data was collected through a self-devised proforma. The Gensini scoring system was used to determine the CAD severity. Participants were stratified into two groups based on the Gensini score system. Statistical analysis was performed using IBM SPSS Statistics for Windows, Version 25 (Released 2017; IBM Corp., Armonk, New York, United States) via an independent t-test and chi-squared tests, Pearson's correlation coefficient, and linear regression analysis. A p-value < 0.05 was set statistically significant.

Results

Of 225 patients, 145 patients (64.45%) had non-severe CAD, while 80 patients (35.55%) had severe CAD. Patients with good glycemic control accounted for n=69 (30.66%), while those with poor glycemic control comprised n=156 (69.34%) of the study population. The differences in means with standard deviations (means ± SD) of Gensini scores (p=0.001) and HbA1c levels (p=0.002) between two study groups (patients with severe CAD and non-severe CAD) were statistically significant. A significant positive correlation was found between HbA1c levels and CAD severity (correlation coefficient (r)=0.75, p=0.002). Moreover, linear regression analysis endorsed the HbA1c level as an important predictor of CAD severity, with a beta coefficient (β) of 3.11 and a 95% CI of 1.52-4.60 (p < 0.001).

Conclusion

This study demonstrates a significant association between HbA1c levels and CAD severity in type 2 diabetic patients with MI. Elevated HbA1c levels are strongly linked to increased CAD severity, highlighting the importance of tight glycemic control in managing CAD in diabetic patients. This study suggests that HbA1c levels can serve as a preliminary marker for early detection of high-risk acute CAD patients, enabling prompt interventions and enhanced clinical outcomes.

## Introduction

Cardiovascular disease is the main cause of morbidity and mortality worldwide, and coronary artery disease (CAD) is its major contributor. According to the World Health Organization (WHO), CAD was responsible for approximately 17.9 million deaths globally in 2019, accounting for 32% of all deaths. Moreover, a major proportion of these deaths took place in developing countries, highlighting the disproportionate burden of CAD in these regions. The estimated number is anticipated to rise to 23.6 million by 2030 [1-3]. In Pakistan, the prevalence of CAD is alarmingly high, with an estimated 18.90% of the population affected by it [2]. In 2019, the age-standardized incidence of cardiovascular disease in Pakistan was also greater than the global average, as per a Global Burden of Disease study [4,5].

The pathophysiology of CAD is characterized by atherosclerosis, an inflammatory condition that leads to the development and progression of atherosclerotic plaques in the coronary arteries. This process is mediated by a complex interplay of inflammatory cells, cytokines, and growth factors, which lead to narrowing and occlusion of the coronary arteries and ultimately result in impaired or intermittent blood supply to the cardiac tissue [6,7]. CAD exhibits diverse clinical manifestations, leading to its categorization into acute coronary syndrome (ACS) and chronic coronary syndromes/stable ischemic heart disease. ACS is further subdivided into three distinct entities: non-ST-elevation myocardial infarction (NSTEMI), unstable angina, and ST-elevation myocardial infarction (STEMI). The most severe manifestation of CAD is acute myocardial infarction (MI), which encompasses both STEMI and NSTEMI [3,8].

Diabetes mellitus (DM) is one of the key risk factors for CAD. A study conducted in the United States revealed that healthcare professionals with diabetes experienced a fourfold increased risk of developing CAD compared to their non-diabetic counterparts [9]. This rise is largely attributed to blood vessel damage resulting from high glucose levels [10]. The connection between DM and CAD is intricate, featuring a two-way interaction where DM boosts CAD risk and CAD worsens DM progression. The pathophysiological mechanisms bridging DM and CAD encompass insulin resistance, inflammation, oxidative stress, and elevated blood glucose levels, which synergistically promote the acceleration of atherosclerotic processes [11-13].

HbA1c is a trusted marker of glycemic control, reflecting average blood glucose levels over the past 2-3 months and serving as a reliable gauge of diabetes management [12,14]. A positive correlation between HbA1c levels and CAD severity has been established through numerous studies that were performed in different settings and mainly in developed countries, highlighting the association between poor glycemic control and elevated cardiovascular risk. Elevated HbA1c levels are indicative of suboptimal glycemic control or hyperglycemia, and hyperglycemia triggers a cascade of deleterious effects, including inflammation, oxidative damage, and vascular injury, thereby increasing the risk and severity of CAD [15-20]. However, the association between HbA1c levels and CAD severity in diabetic patients who have had an MI is not well-defined, particularly in developing countries where healthcare resources may be limited [10,21,22].

In Pakistan, there is a lack of studies investigating the association between HbA1c levels and CAD severity in diabetic patients with MI, which highlights the need for research in this area to better understand the relationship between glycemic control and cardiovascular risk in this high-risk population. Therefore, this study aims to investigate the association between glycated hemoglobin (HbA1c) and the severity of CAD among type 2 diabetic patients with MI. This study will provide valuable insights into the relationship between glycemic control and cardiovascular risk in diabetic patients with MI. Moreover, the findings of this study will also have important implications for clinicians, policymakers, and researchers and will contribute to the development of effective strategies for the prevention and effective management of CAD in diabetic patients.

## Materials and methods

Study design and study population

This cross-sectional study was conducted at the Cardiology Unit of Jinnah Hospital, Lahore, Pakistan, over a one-year period from August 2020 to August 2021. A total of 225 diabetic patients with acute MI who underwent coronary angiography were selected through consecutive sampling (to reduce selection bias), based on predefined inclusion and exclusion criteria. The sample size was calculated using the OpenEpi sample size calculator, by using a prevalence of 17.50% of coronary heart disease and a similar effect size from a study by Zubair et al., a 95% confidence interval, a 5% margin of error, and an 80% power of study [1]. Ethical approval from the ethical review board of Jinnah Hospital and informed consent from the patients were obtained before the start of the study.

Inclusion and exclusion criteria

The study included patients of both genders, aged thirty years or older, with diagnosed type 2 diabetes mellitus, a complete medical record of ACS confirmed through electrocardiography (ECG), cardiac biomarkers, and coronary angiography. Conversely, patients under thirty years old and with a history of previous ischemic heart disease, heart failure (HF), congenital heart disease, chronic liver or kidney disease, anemia, autoimmune disorder, active infection, malignancy, and recent surgeries (within the last three months) were excluded from the study. The exclusion criterion was applied to rule out potential confounding factors, such as chronic kidney disease, HF, valvular disease, and metabolic disorders, ensuring that any observed ECG changes were solely attributed to ACS rather than these underlying conditions.

Primary outcomes and secondary outcomes

The primary objective of this study was to examine the relationship between HbA1c levels and the severity of CAD, as evaluated by the Gensini Scoring System. In addition to this primary aim, the study also explored three secondary outcomes. Firstly, the study compared HbA1c levels between two distinct patient cohorts: those with severe CAD and those with non-severe CAD. Secondly, to investigate the potential utility of HbA1c as a prognostic indicator, the study assessed its predictive value in determining CAD severity. Thirdly, to analyze the association between the severity of CAD and traditional cardiovascular risk factors. This study hypothesized that higher HbA1c levels are associated with increased CAD severity. The clinical implications of this study are to explore the potential utility of HbA1c as a biomarker for CAD risk stratification and to inform the development of personalized treatment strategies for patients with CAD.

Assessment of study parameters

The diagnosis of MI was established in accordance with the American Heart Association's criteria, which required a combination of severe chest pain lasting at least 30 minutes, distinctive ECG patterns indicative of MI, and significant elevations in cardiac enzymes [3]. CAD was diagnosed by coronary angiography as per the American College of Cardiology/American Heart Association lesion classification guidelines, the presence of severe stenosis at least 50% of vessel diameter in any primary coronary artery, was regarded as CAD. The Gensini scoring system was applied to determine the CAD severity. The Gensini score is a globally recognized angiographic grading system used to assess the severity of CAD. It was calculated in accordance with the established Gensini score criteria. The calculation involves three key steps: first, determining the severity score of the coronary artery lesion based on a percentage of stenosis in the vessel; second, applying a multiplication factor to each lesion score based on its anatomical position within the coronary vasculature; and third, summing up lesion severity scores to obtain the final Gensini score. Based on the Gensini scores, patients with coronary artery stenosis were divided into two categories: patients with a Gensini score up to 50 were categorized to have non-severe CAD, whereas patients with a Gensini score more than 50 were marked as having severe CAD. To address the inter-observer variability in the angiographic assessment, the Gensini score was independently validated by two experienced cardiologists, using a standardized protocol [3,6]. At admission of the patients, levels of serum lipids and HbA1c were measured from blood samples, and patients were split into groups based on their HbA1c levels: patients with good glycemic control (HbA1c level below 7.5%) and poor glycemic control (HbA1c level above 7.5%). High-performance liquid chromatography with a certified laboratory kit was used for the measurement of HbA1c levels [10].

Data collection tool

Data collection was done through a pre-tested self-devised proforma (Table 4 in Appendices). It had two parts. The first part focused on medical history and physical examination findings, including factors such as age, sex (male or female), and the presence or absence of traditional risk factors for CAD, such as family history of CAD, hypertension, dyslipidemia, and history of smoking. The second part was related to the results of all diagnostic tests. All lab tests including levels of cardiac biomarkers, HbA1c, serum lipid, and other relative investigations like ECG and coronary angiography, were carried out at JH during the management of patients.

Data analysis

Statistical analysis was performed using IBM SPSS Statistics for Windows, Version 25 (Released 2017; IBM Corp., Armonk, New York, United States). We applied parametric statistical tests to analyze the data, as the Shapiro-Wilk test confirmed that the data followed a normal distribution. Frequencies and percentages were utilized to illustrate nominal data, while numerical data were presented through mean ± standard deviation. Quantitative and qualitative parameters were compared between the two study groups by applying the independent t-test and chi-squared tests, respectively. The association between Gensini scores and the HbA1c levels was determined using Pearson's correlation analysis. Moreover, the predictive value of HbA1c for Gensini scores was assessed via a linear regression model. P-values < 0.05 were viewed as statistically significant.

## Results

Among the 225 patients, non-severe CAD was identified in 145 patients (64.45%), whereas severe CAD was found in 80 patients (35.55%). The frequency of patients with good glycemic control and poor glycemic control was n=69 (30.66%) and n=156 (69.34%), respectively. 

Table 1 indicates the study population's demographic and clinical characteristics. It also shows significant variations between the two study groups (non-severe CAD group and severe CAD group) in two key variables, including Gensini score and HbA1c levels, with p-value < 0.05. Moreover, although the frequency of severe CAD was higher among the patients with male gender, positive family history of CAD, hypertension, dyslipidemia, and positive history of smoking, however, no statistically significant difference was observed in the mean of age and frequency of gender, family history of CAD, hypertension, dyslipidemia, and history of smoking, between the two study groups (p > 0.05). 

**Table 1:** Demographic and clinical features of the study population along with independent t-test and chi-squared test analysis N, study population size; n, sample size for each group or category; %, percentage; SD, standard deviation. The table presents test statistic values and p-values from two statistical tests, distinguished by symbols. Values marked with a "*" sign represent the results of independent t-tests, while those marked with a "+" sign represent the results of chi-squared tests. This notation applies to both the test statistic values in the second-to-last column and the corresponding p-values in the last column.

Variables with Myocardial Infarction N=225		Expression of Variables	Severity of Coronary Artery Disease		Chi-Squared test/Independent t-test	
					Test Statistics	
			Non-severe Coronary Artery Disease Group n=145 (64.45%)	Severe Coronary Artery Disease Group n=80 (35.55%)	χ²-value for Chi-Square test/ t-value for Independent-test	p-values
Age (Years) (Means ± SD)		58.77±18.24	57.87±14.65	59.76±19.80	1.84^*^	0.06^*^
Gensini Score (Means ± SD)		49.48 ±34.86	29.81±16.29	72.60±35.49	12.15^*^	0.001^*^
HbA1c Level (Means ± SD)		10.18±2.78	8.40±1.20	11.39±3.27	8.52^*^	0.002^*^
Gender	Male n (%)	135 (60.00)	89 (61.38)	46 (57.50)	0.38^+^	0.54^+^
	Female n (%)	90 (40.00)	56 (38.62)	34 (42.50)		
Family History of Coronary Disease	Yes n (%)	78 (34.66)	33 (22.75)	45 (56.25)	3.41^+^	0.064^+^
	No n (%)	147 (65.37)	112 (77.25)	35 (43.75)		
Hypertension	Yes n (%)	166 (73.77)	114 (78.62)	52 (65.00)	0.54^+^	0.46^+^
	No n (%)	59 (26.23)	31 (21.38)	28 (35.00)		
Dyslipidemia	Yes n (%)	128 (56.88)	85 (58.62)	43 (53.75)	0.53^+^	0.47^+^
	No n (%)	97 (43.12)	60 (41.38)	37 (46.25)		
History of Smoking	Yes n (%)	101 (44.89)	48 (33.10)	53 (66.25)	1.53^+^	0.21^+^
	No n (%)	124 (55.11)	97 (66.90)	27 (33.75)		

Table 2 reveals a significant strongly positive association between HbA1c levels and the severity of CAD, as evidenced by Pearson's correlation analysis, within the study cohort. This significant correlation demonstrates that as the HbA1c level rises, the Gensini scores also exhibit a tendency to rise, signifying a direct correspondence between the HbA1c levels and the CAD severity.

**Table 2:** Correlation between glycated hemoglobin levels and the severity of coronary artery disease in the study population HbA1c, glycated hemoglobin

Variables of Patients with Myocardial Infarction N=225	Severity of Coronary Artery Disease		Independent t test		Pearson’s Correlation	
			Test Statistics		Test Statistics	
	Non-severe Coronary Artery Disease Group	Severe Coronary Artery Disease Group	t-value	p-value	Correlation Coefficient (r)	p-value
Gensini Score	29.81±16.29	72.60±35.49	12.15	0.001	0.75	0.002
HbA1c Levels	8.40±1.20	11.39±3.27	8.52	0.002		

Table 3 shows that the simple linear regression model was significantly fit (R² = 0.81, p = 0.000), demonstrating a statistically significant positive correlation between HbA1c levels and Gensini scores. The positive beta coefficient suggests that elevated HbA1c levels are strongly linked to increased Gensini scores, indicating a higher severity of CAD.

**Table 3:** Assessment of predictive value of glycated hemoglobin levels for the severity of coronary artery disease via a simple linear regression model CI, confidence interval; HbA1c, glycated hemoglobin

Variable	Test Statistics for Simple Linear Regression Model				
	Unstandardized Regression Coefficient (β)	95% CI	p-value	R^2 ^value	p-value of F test
HbA1c Levels	3.11	1.52 to 4.60	0.001	0.81 (81.00%)	0.000

## Discussion

Cardiovascular disease is the leading cause of mortality all over the world, while CAD contributes its major part. One of the major risk factors of CAD is diabetes mellitus. Therefore, it is crucial to investigate the association between CAD and diabetes mellitus particularly in developing countries, as in these countries, research on this association is limited [1,2,21,22]. In the present investigation, we have garnered crucial insights into the relationship between HbA1c and the severity of CAD. Additionally, we have also noted the prevalence of various traditional risk factors contributing to CAD, as well as variations in the distribution of these risk factors between two distinct study cohorts: patients with non-severe CAD and those with severe CAD. 

In the present study, a total of 225 patients were analyzed, revealing that 145 (64.45%) exhibited non-severe CAD, whereas 80 (35.55%) presented with severe CAD. Interestingly, a higher prevalence of severe CAD has been observed in the Indian population with a similar risk factor profile [16]. Regarding the glycemic control in the study, the distribution of patients with good and poor glycemic control was 30.66% (n=69) and 69.34% (n=156), respectively. A similar distribution of patients based on glycemic control has been presented in another study among diabetic patients with CAD [10]. The clinical significance of this finding of a high proportion of patients with poor glycemic control emphasizes the need for aggressive diabetes management strategies to improve glycemic control and mitigate the risk of cardiovascular events in patients with diabetes.

Analysis of the demographic characteristics of the study population revealed that the average age of patients with severe CAD (59.76 years ± 19.80 SD) was higher than that of patients with non-severe CAD (57.87 years ± 14.65 SD). Furthermore, CAD frequency was greater among men (n=130; 60.00%). These findings have been supported by another study, in which similar demographic trends among patients with CAD had been noted [6].

Although the prevalence of severe CAD was higher among patients with a positive family history of CAD, hypertension, dyslipidemia, and smoking history; however, the difference was statistically insignificant as compared to patients without these risk factors. In the diabetic type 2 study population, the most common traditional risk factor was hypertension, followed by dyslipidemia, history of smoking, and positive family history of CAD. The same pattern of these factors has been observed across different studies [1,6,11].

The clinical diagnostic power of HbA1c has been demonstrated in various studies, including a recent evaluation of incidental diabetes mellitus in patients with hyperglycemia in the emergency department, which highlighted the utility of HbA1c in diagnosing diabetes [19,18,23]. In the present investigation, as per correlation coefficient (r = 0.75) and regression coefficient (β = 3.11), a strongly positive and significant correlation was observed between HbA1c levels and CAD severity (Gensini scores), wherein patients with higher CAD severity exhibited elevated HbA1c levels. This primary finding is consistent with numerous preceding studies conducted globally. A retrospective cohort study of China among 300 participants, also demonstrated similar results that HbA1c levels are significantly associated with CAD severity [6]. Furthermore, another cross-sectional study among 151 patients highlighted the association between elevated HbA1c levels and increased CAD severity among type 2 diabetic patients [10]. A Turkish retrospective study that was performed among 247 patients has also yielded consistent findings regarding the relationship between HbA1c levels and CAD severity, revealing that an increase in serum HbA1c levels corresponds to a rise in CAD severity [14]. An Indian prospective study among 208 patients has also reported analogous observations, demonstrating that elevated serum HbA1c levels are associated with increased CAD severity [16]. Consistent with these findings, a meta-analysis that included studies with prospective cohort and case-control study designs has shown a significant correlation between serum HbA1c levels and CAD severity, mirroring the results of the present study [17]. Furthermore, a Brazilian cohort study among 888 patients has noticed that patients with higher HbA1c levels tend to have more severe CAD compared to those with lower HbA1c levels [18]. An Australian study with 1187 participants has likewise observed fluctuations in HbA1c levels across various patient groups with differing CAD severities [19]. Furthermore, a 10-year longitudinal cohort study among 1302 participants has corroborated the findings of the present investigation [20]. Most of these comparative studies used correlation and regression analysis to assess the association between HbA1c levels and CAD severity, and they found statistically significant results. The results of this study advocate for the utilization of serum HbA1c levels as an important biomarker for assessing CAD severity in type 2 diabetic patients experiencing myocardial infarction, aligning with the conclusions of previous research.

The relationship between HbA1c levels and CAD severity can be attributed to multiple underlying mechanisms. High HbA1c levels are indicative of suboptimal glucose control, which can trigger a cascade of events including inflammation, oxidative damage, and vascular injury, thereby increasing the likelihood of CAD development [11,12]. Furthermore, hyperglycemia can initiate several pathways that contribute to the initiation and progression of atherosclerotic disease, including the activation of protein kinase C, the accumulation of advanced glycosylation end-products, and the upregulation of pro-inflammatory cytokines. Moreover, these pathways also contribute to plaque instability, increasing the risk of acute cardiovascular events [13-15].

The study's findings have significant implications for the management of CAD in diabetic patients. The study suggests that HbA1c levels can serve as a useful tool for the stratification of diabetic patients with CAD by presenting their glycemic control status. It could help for early detection of high-risk acute CAD patients, enabling prompt interventions and enhanced clinical outcomes. Additionally, the study highlights the importance of tight glycemic control in managing CAD in diabetic patients. This can be achieved through a combination of lifestyle modifications, such as diet and exercise, and pharmacological interventions, such as oral hypoglycemic drugs and insulin therapy. The use of HbA1c levels as a marker along with existing CAD risk scores, for early detection and risk stratification, can enable healthcare providers to initiate personalized treatment strategies, which can lead to improved clinical outcomes and reduced mortality rates. Furthermore, the study's findings have significant implications for healthcare policy, as policymakers can use the study's findings to develop guidelines and protocols for the management of CAD in diabetic patients.

Although to minimize potential confounding effects and ensure a more homogeneous study population, we excluded patients with pre-existing chronic diseases. This exclusion was necessary to focus on the relationship between HbA1c levels and CAD severity in patients without pre-existing chronic diseases, which may have different underlying pathophysiological mechanisms. However, this investigation has several limitations. The modest sample size and single-center design may limit the generalizability of our findings. The cross-sectional design precludes establishing causality between HbA1c levels and CAD severity. Additionally, unadjusted potential confounders may have impacted our results. To address these limitations, further research is necessary. Specifically, studies with prospective designs and randomized controlled trials are needed to validate our findings, establish causality, and elucidate the underlying mechanisms driving the association between HbA1c levels and CAD severity. Future studies should also consider adjusting for potential confounders using statistical methods, such as multivariate regression analysis, to provide a more accurate estimate of the relationship between HbA1c levels and CAD severity. Multicenter studies conducted in diverse geographic locations will also help to increase the generalizability of our findings.

## Conclusions

This study has shown that patients with severe CAD had elevated HbA1c levels compared to those with non-severe CAD. A significant strong positive correlation was found between HbA1c levels and CAD severity, as evaluated by the Gensini scoring system. Notably, HbA1c calculation is a straightforward and economically viable process. Consequently, its inclusion as a preliminary risk stratification tool during cardiac risk assessment is advocated, particularly in healthcare facilities (primary care or resource-limited hospitals) lacking advanced diagnostic tools like coronary angiography. Our research underscores the importance of prompt and adequate management of cardiac patients with higher HbA1c levels by cardiologists, as they are more likely to have severe CAD compared to those with lower HbA1c levels. Clinicians are advised to consider HbA1c levels in conjunction with other diagnostic modalities and cardiac assessment scoring systems for a comprehensive CAD evaluation. Integrating HbA1c level assessment into clinical practice may yield enhanced patient outcomes and reduced mortality rates among CAD patients. Although the present study suggests the use of HbA1c levels in conjunction with other cardiac diagnostic investigations and scoring systems, however, it also acknowledges the need for prospective validation before HbA1c can be routinely integrated into CAD risk assessment protocols.

## Appendices

**Table 4:** Research proforma for association between glycated hemoglobin and severity of coronary artery disease in type 2 diabetic patients with myocardial infarction HbA1c, glycated hemoglobin

Sections	Research Questions	Options: write/tick the option	
Section A. (History, Physical Examination, and Medical Records)			
1. What was the age of the patient? (Years)			
2. What was the gender of the patient?		Male	Female
3. What were the presenting complaints of the patient?			
4. What was the duration of presenting complaints? (Minutes)			
5. Presence of family history of coronary artery disease?		Yes	No
6. Previous history of diabetes mellitus?		Yes	No
7. Previous history of hypertension?		Yes	No
8. Previous history of dyslipidemia?		Yes	No
9. Previous history of smoking?		Yes	No
10. Previous history of coronary artery disease/cardiac or any other surgery?		Yes	No
11. Previous treatment history?		Yes	No
12. Presence of any chronic disease other than that are mentioned above?		Yes	No
13. What were the physical examination findings of the patient? (Vitals, General, and Systemic)			
14. What were the findings of previous medical record?			
Section B. (Investigations Reports)			
1. Electrocardiogram findings			
2. Cardiac biomarker levels (Troponin I) (ng/ml)			
3. Coronary angiography findings			
4. Gensini Score			
5. Severity of coronary artery disease based on the Gensini Score	Non-severe Coronary Artery Disease Group (Up to 50)	Severe Coronary Artery Disease Group (Above 50)	
6. HbA1c level (%)			
7. Glycemic status based on HbA1c Levels	Good glycemic control (less than 7.5%)	Poor glycemic control (above 7.5%)	
8. Serum lipid level (mg/dL)			
9. Lipid level status?	Normal (less than 200 mg/dL)	Abnormal (above 200 mg/dL)	
10. Was there any abnormality in the liver function tests/renal function test/C-reactive protein/erythrocyte sedimentation rate/white blood cell count)?	Yes	No	
